# Supplementary material for: Antimicrobial Resistance Profile of Bacterial Isolates from Urinary Tract Infections in Companion Animals in Central Italy
Source: Antibiotics (Basel). 2022 Oct 6;11(10):1363. doi: 10.3390/antibiotics11101363 (PMC9598067; doi:10.3390/antibiotics11101363)
Supplement: Supplementary file 1 [file antibiotics-11-01363-s001.zip › antibiotics-1945951-supplementary.pdf]

Table S1. Table summarizes the resistant bacteria isolated from urine samples in dogs

Antibiotics	*Acinetobacter* spp. n=1	*E. coli* n=263	*Enterobacter* spp. n=13	*Enterococcus* spp. n=30	*Klebsiella* spp. n=21	*Pantoea dispersa* n=1	*Pausterella pneumotropica* n=2	*Proteus mirabilis* n=86	*Pseudomonas* spp. n=18	*Raoultella ornithinolytica* n=1	*Staphylococcus* spp. n=14
Ampicillin	1(100%)	125(47.5%)	-	-	21(100%)	-	0	86 (100%)	17 (94.4%)	1 (100%)	-
Amoxicillin/Clavulanic acid	-	46(17.4%)	13(100%)	12(40%)	2(9.5%)	0	-	2 (2.32%)	-	1 (100%)	3(21.4%)
Amikacin	-	0	0	-	0	0	-	7 (8.13%)	0	0	0
Chloramphenicol	-	29(11%)	4(30.7%)	3(10%)	4(19%)	0	-	41 (47.6%)	-	1 (100%)	3(21.4%)
Cephalothin	-	101(38.4%)	13(100%)	-	7(33.3%)	0	-	41 (47.6%)	-	1 (100%)	2(14.2%)
Ceftiofur	1(100%)	53(20.1%)	8(61.5%)	-	7(33.3%)	0	0	27 (31.3%)	16 (88.8%)	1 (100%)	3(21.4%)
Cephalexin	1(100%)	82(31.1%)	13(100%)	-	7(33.3%)	0	0	41 (47.6%)	17 (94.4%)	1 (100%)	0
Cefpodoxime	1(100%)	81(30.7%)	10(76.9%)	-	7(33.3%)	0	0	40 (46.5%)	17 (94.4%)	1 (100%)	2(14.2%)
Doxycycline	0	47(17.8%)	4(30.7%)	17(56.6%)	4(19%)	0	-	74 (86%)	1 (5.5%)	1 (100%)	2(14.2%)
Enrofloxacin	-	33(12.5%)	7(53.8%)	14(46.6%)	7(33.3%)	0	-	19 (22%)	5 (27.7%)	1 (100%)	5(35.7%)
Nitrofurantoin	1(100%)	53(20.1%)	4(30.7%)	4(13.3%)	11(52.3%)	0	0	86 (100%)	17 (94.4%)	0	4(28.5%)
Gentamicin	0	3(1.14%)	4(30.7%)	-	0	0	-	13 (15.1%)	1 (5.5%)	1 (100%)	1(7.1%)
Imipenem	0	2(0.7%)	1(7.6%)	-	0	0	0	51 (59.3%)	1 (5.5%)	0	-
Marbofloxacin	0	6(2.28%)	8(61.5%)	15(50%)	7(33.3%)	0	0	13(15.1%)	2 (11.1%)	0	5(35.7%)

Trimethoprim/Sulfamethoxazole	0	8(3%)	8(61.5%)	-	3(14.2%)	1(100%)	0	31(36%)	16 (88.8%)	1 (100%)	4(28.5%)
Tetracycline	1(100%)	9(3.4%)	3(23%)	10(33.3%)	3(14.2%)	1(100%)	0	86(100%)	16 (88.8%)	1 (100%)	3(21.4%)
Cefovecin	1(100%)	5(1.9%)	8(61.5%)	30(100%)	7(33.3%)	0	0	24(27.9%)	14 (77.7%)	1 (100%)	5(35.7%)
Neomycin	-	2(0.7%)	0	-	0	0	0	1(1.16%)	-	0	1(7.1%)
Pradofloxacin	-	6(2.28%)	8(61.5%)	-	7(33.3%)	0	0	16(18.6%)	-	1 (100%)	5(35.7%)
Florfenicol	-	-	-	1(3.33%)	-	-	-	-	-	-	0
Benzylpenicillin	-	-	-	12(40%)	-	-	-	-	-	-	12(85.7%)
Minocycline	-	-	-	5(16.6%)	-	-	-	-	-	-	0
Erythromycin	-	-	-	17(56.6%)	-	-	-	-	-	-	10(71.4%)
Clindamycin	-	-	-	-	-	-	-	-	-	-	8(57.1%)
Oxacillin	-	-	-	-	-	-	-	-	-	-	7(50%)
Kanamycin	-	-	-	-	-	-	-	-		-	1(7.1%)

Table S2. Table summarizes the resistant bacteria isolated from urine samples in cats

**Antibiotics**	***Acinetobacter* spp. n=2**	***E. coli* n=80**	***Enterobacter* spp. n=10**	***Enterococcus* spp. n=40**	***Klebsiella* spp. n=8**	***Pantoea dispersa* n=1**	***Proteus mirabilis* n=2**	***Pseudomonas aeruginosa* n=1**	***Staphylococcus* spp. n=11**
Ampicillin	1(50%)	44(55%)	-	-	8(100%)	-	2(100%)	1(100%)	-
Amoxicillin/Clavulanic acid	-	12(15%)	10(100%)	4(10%)	4(50%)	0	-	-	5(45.4%)
Amikacin	0	0	0	-	0	0	0	0	0
Chloramphenicol	-	9(11.2%)	6(60%)	6(15%)	3(37.5%)	0	2(100%)	-	4(36.3%)
Cephalothin	-	34(42.5%)	10(100%)	-	7(87.5%)	0	0	-	3(27.2%)
Ceftiofur	2(100%)	18(22.5%)	9(90%)	-	7(87.5%)	0	0	1(100%)	4(36.3%)
Cephalexin	1(50%)	25(31.2%)	9(90%)	-	7 (87.5%)	0	0	1(100%)	-
Cefpodoxime	2(100%)	28 (35%)	8(80%)	-	7(87.5%)	0	0	1(100%)	1(9%)
Doxycycline	0	13(16.2%)	2(20%)	17(42.5%)	6(75%)	0	0	-	1(9%)
Enrofloxacin	-	17(21.2%)	8(80%)	14(35%)	8(100%)	0	2(100%)	1(100%)	6(54.5%)
Nitrofurantoin	1(50%)	15(18.7%)	1(10%)	0	3(37.5%)	0	2(100%)	1(100%)	1(9%)
Gentamicin	1(50%)	4(5%)	4(40%)	-	0	0	0	0	2(18.1%)
Imipenem	1(50%)	0	0	-	0	0	1(50%)	0	-
Marbofloxacin	1(50%)	17(21.2%)	-	21(52.5%)	-	0	2(100%)	0	-
Trimethoprim/Sulfamethoxazole	0	18(22.5%)	7(70%)	-	6(75%)	0	2(100%)	1 (100%)	5 (45.4%)
Tetracycline	1(50%)	19(23.7%)	5(50%)	8(20%)	7(87.5%)	0	2(100%)	1 (100%)	2 (18.1%)
Cefovecin	2 (100%)	11(13.7%)	9(90%)	38(95%)	6(75%)	0	0	1 (100%)	5 (45.4%)

Neomycin	-	5(6.25%)	0	6(15%)	0	0	0	-	1(9%)
Pradofloxacin	-	17(21.2%)	8(80%)	-	6(75%)	0	2(100%)	1(100%)	6(54.5%)
Lincomycin	-	-	-	-	-	-	-	-	-
Erythromycin	-	-	-	17(42.5%)	-	-	-	-	6(54.5%)
Minocycline	-	-	-	11(27.5%)	-	-	-	-	-
Benzylpenicillin	-	-	-	3(7.5%)	-	-	-	-	6(54.5%)
Florfenicol	-	-	-	-	-	-	-	-	1(9%)
Kanamycin	-	-	-	-	-	-	-	-	4(36.3%)
Oxacillin	-	-	-	-	-	-	-	-	5(45.4%)
Clindamycin	-	-	-	-	-	-	-	-	5(45.4%)
